# Epidermal growth factor receptor cascade prioritizes the maximization of signal transduction

**DOI:** 10.1038/s41598-022-20663-0

**Published:** 2022-10-10

**Authors:** Kaori Kiso-Farnè, Tatsuaki Tsuruyama

**Affiliations:** 1 Center for anatomical, pathological, and forensic medical researches, Graduate School of Medicine, Kyoto University, Kyoto, 606-8501 Japan; 2 Drug and Discovery Medicine, Graduate School of Medicine, Medical Innovation Center, Kyoto University, Kyoto, 606-8507 Japan; 3 Department of Physics, Graduate School of Science, Tohoku University, Aramaki, Aoba-ku 6-3, Sendai, 980-8578 Japan; 4 Department of Molecular Biosciences, Radiation Effects Research Foundation, Minami-ku, Hiroshima, 732-0815 Japan; 5 Department of Tumor Research, Kitano Hospital, The Tazuke Kofukai Medical Research Institute, Kita-ku, Osaka, 530-8480 Japan

**Keywords:** Computational biophysics, Molecular biophysics

## Abstract

Many studies have been performed to quantify cell signaling. Cell signaling molecules are phosphorylated in response to extracellular stimuli, with the phosphorylation sequence forming a signal cascade. The information gain during a signal event is given by the logarithm of the phosphorylation molecule ratio. The average information gain can be regarded as the signal transduction quantity (ST), which is identical to the Kullback–Leibler divergence (KLD), a relative entropy. We previously reported that if the total ST value in a given signal cascade is maximized, the ST rate (STR) of each signaling molecule per signal duration (min) approaches a constant value. To experimentally verify this theoretical conclusion, we measured the STR of the epidermal growth factor (EGF)-related cascade in A431 skin cancer cells following stimulation with EGF using antibody microarrays against phosphorylated signal molecules. The results were consistent with those from the theoretical analysis. Thus, signaling transduction systems may adopt a strategy that prioritizes the maximization of ST. Furthermore, signal molecules with similar STRs may form a signal cascade. In conclusion, ST and STR are promising properties for quantitative analysis of signal transduction.

## Introduction

Signal transduction systems are unique chain reactions involving signaling molecules in biological systems. A well-known example is the epidermal growth factor (EGF)-driven signal cascade in cancer cells^1^. Many pioneering studies recently reported that information science theory is a powerful framework for quantitatively understanding signal transduction^2^. Using this framework allows researchers to identify the information transmission strategies used by cells^3–5^.

We have recently been developing a method for quantifying signal transduction involving the Kullback–Leibler divergence (KLD), a relative entropy that expresses the average difference in information entropy before and after a signal event. The difference is called information gain^6–8^. The KLD has also been used in other fields, such as clinical trial research^9^, Bayesian model diagnostics^10^, and bioequivalence evaluations^11^.

A431 skin cancer cells can be used to evaluate EGF signaling. Stimulating the EGF receptor (EGFR) of the cultured cells causes sequential phosphorylation of Raf1, mitogen-activated protein kinase (MAPK)-extracellular signal-regulated kinase 1 (MEK1), and kinase-extracellular signal-regulated kinase 1 (ERK1), which allows for the transmission of information along with other transcription factors into the nucleus, followed by cell proliferation^12–19^. This sequential phosphorylation of signaling molecules is considered to form the Raf1-MEK1-ERK1 signaling cascade^1^. In addition, the MAPK cascade is essential for cancer cell proliferation^14–17^. In the absence of EGFR stimulation, signaling molecules are constitutively phosphorylated through other types of stimulation. Therefore, the phosphorylation level at the time of signal transduction by EGF receptor stimulation should be evaluated as the difference between the post- and pre-stimulation values. The KLD is an information gain, representing the average value of the difference in information entropy before and after signal transduction; therefore, the KLD is an appropriate approach for quantitatively evaluating differences before and after receptor stimulation. This study utilized the KLD to quantify signal transduction in the Raf1-MEK1-ERK1 and MAPK signaling cascades in A431 cells.

## Results

### Signal transduction model

First, we consider the signal cascade consisting of *n* steps in which the signal molecules are phosphorylated. The *j-*th molecule *X*_*j*_ denotes the signal molecule that is phosphorylated at the *j-*th step (1 ≤ *j* ≤ *n*). For example, in Raf1-MEK1-ERK1, the 1st, 2nd, and 3rd molecules are Raf1, MEK1, and ERK1, respectively. The initial proportion of the *j-*th signal molecules participating in signal transduction before EGF stimulation is expressed as follows:

1 $$p_{j}^{st} = X_{j}^{st} /X,$$

where *X* (= Σ_*j* =1_^*n*^* X*_*j*_) denotes the sum of signal molecules, and the superscript *st* denotes the stable state before a signal event. The proportion after EGF stimulation is expressed as follows:

2 $$p_{j} \left( t \right) = X_{j} \left( t \right)/X$$

**Figure 1:**
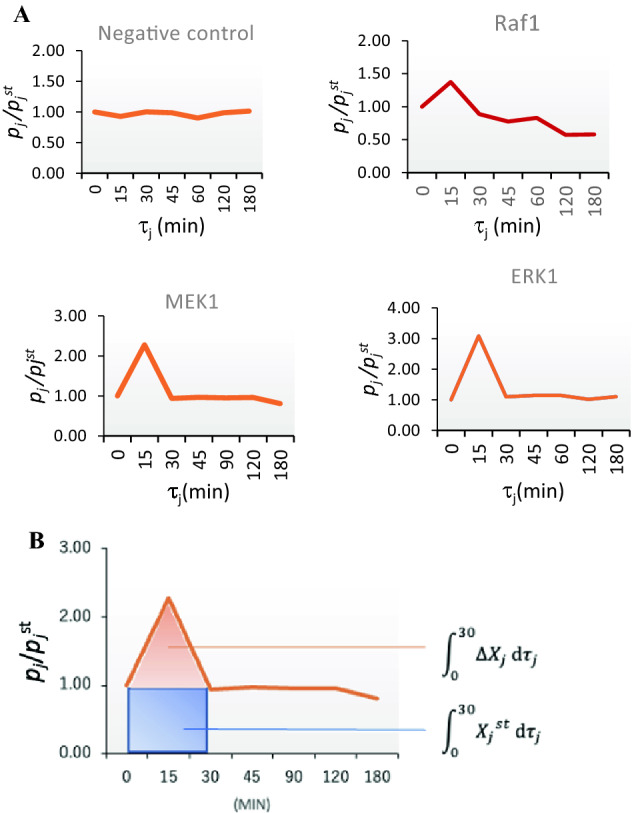
(**A**) Time course of the phosphorylation of signaling molecules in starved A431 cells. The ratio *p*_*j*_/*p*_*j*_^*st*^ = *X*_*j*_/*X*_*j*_^*st*^ is shown on the *y*-axis, and the time after epidermal growth factor (EGF) stimulation (min) is shown on the *x*-axis. Plots of actual experimental time-course data are provided (S1 Data and S2 Data). The negative control, Raf1, MEK1, and ERK1 plots represent the ratio intensity. The typical time course pattern indicates that the phosphorylation of one signal molecule increases from *t* = 5 to 15 min after stimulation and declines from *t* = 15 to 45 min. (**B**) Time course of phosphorylation of the signal molecule *X*_*j*_. Information entropy gain *ΔI*_*j*_ (= log *p*_*j*_/*p*_*j*_^*st*^) was calculated using the ratio ∫_0_${^{\tau _{j}}}$ (*ΔX*_*j*_ + *X*_*j*_^*st*^) *dt*/∫_0_${^{\tau _{j}}}$*X*_*j*_^*st*^* dt*. In this case, *τ*_*j*_ = 30 min.

*X*_*j*_(*t*) and *p*_*j*_(*t*) are the functions of time *t* during the signal event, and *t* = 0 represents the time for which the cell is stimulated. The increases in ∆*X*_*j*_(*t*) = *X*_*j*_(*t*) - *X*_*j*_^st^ and Δ*p*_*j*_(*t*) (= *p*_*j*_(*t*) − *p*_*j*_^*st*^) depend on the amount of time that has passed, and plots of the actual experimental time-course data are provided (S1 Data). The typical time course pattern of *p*_*j*_(*t*) (and *X*_*j*_) increases and then decreases to *p*_*j*_^*st*^ during *τ*_*j*_ (Fig. 1A)^20^. This pattern indicates that the phosphorylation of one signal molecule increases within the first few minutes after stimulation and declines to the initial level within 3 h (Fig. 1A). Subsequently, we designated the average signal duration of the *j*th signal molecule *X*_*j*_ as *τ*_*j*_, which indicates the duration from the increase in the phosphorylated signal to the decline to the initial phosphorylation level of the molecule. Using *τ*_*j*_, total signal event duration *τ* is defined as the sum of each phosphorylation duration multiplied by the molecule concentration.

3 $$\begin{array}{c}\tau = {\sum }_{j=1}^{n}{X}_{j} {\tau }_{j} =X{\sum }_{j=1}^{n}{p}_{j} { \tau }_{j} \end{array}$$

Information entropy is expressed as *I*_*j*_ = − log *p*_*j*_, and the information entropy gain during the signal event is denoted as Δ*I*_*j*_ = log *p*_*j*_ − log *p*_*j*_^*st*^ = log *p*_*j*_/*p*_*j*_^*st*^. The average information entropy gain in a given signal cascade is expressed as Σ_*j*_* p*_*j*_ log *p*_*j*_/*p*_*j*_^*st*^^7^. This form is regarded as the signal transduction quantity (ST), which is identical to the KLD, a relative entropy.

In the current study, we evaluated the EGFR signal cascade comprising the chain of phosphorylation reactions of Ras, Raf1, MEK1, and ERK1. Based on the definition above, the signal amount of the EGFR cascade was defined using the KLD^4,5,21^. For example, when Raf1 has a signal value of − log *p*_*Raf1*_^*st*^ before the signal event, − log *p*_*Raf1*_ changes in a time-dependent manner following EGFR stimulation. In this case, the signal information entropy gain for Raf1 phosphorylation was determined by calculating the log (*p*_*Raf1*_/*p*_*Raf1*_^*st*^). Therefore, ST of Raf1-MEK1-ERK1 was calculated in the form of the KLD^2,7^:

4 $$\begin{array}{c}\Delta I=X\sum_{j=1}^{n}{p}_{j}\Delta {I}_{j}=X\sum_{j}{p}_{j}\mathrm{log}\frac{{p}_{j}}{{{p}_{j}}^{st}} =\sum_{j}{X}_{j}\mathrm{log}\frac{{X}_{j}}{{{X}_{j}}^{st}}\end{array}$$

where *j* = Raf1, MEK1, and ERK1. $\Delta I$ represents the expected ST and *I*_*j*_ = log (*p*_*j*_/*p*_*j*_^st^).

### Signal transduction maximization and signal transduction rate independent of signal molecule type

The equation above was obtained from Eqs. (1)–(3); the total signal transduction rate was expressed as $\Delta I$/$\tau$ and each signal transduction rate (STR) as $\Delta {I}_{j}$/${\tau }_{j}$. Then, the STR of the *j-*th signal molecule was expressed as follows^7^:

5 $$\begin{array}{c}\frac{\Delta {I}_{j}(0\le t\le {\tau }_{j})}{{\tau }_{j}}=\frac{1}{{\tau }_{j}}\mathrm{log}\frac{{p}_{j}(0\le t\le {\tau }_{j})}{{{p}_{j}}^{st}}= \frac{1}{{\tau }_{j}}\mathrm{log}\frac{{\int }_{0}^{{\tau }_{j}}{{X}_{j}}^{st}+\Delta {X}_{j}dt}{{\int }_{0}^{{\tau }_{j}}{{X}_{j}}^{st}dt}={\beta }_{j}\end{array}$$

The integrals in the third item in Eq. (5) were calculated using the integral of the plot area (Fig. 1B). The STR of each molecule, *β*_*j*_, should vary for each signal molecule. However, we previously reported that signal molecules in the same cascade yield identical STR values when the ST is maximized^21^; this is discussed in the subsequent paragraph.

We assumed that the EGFR signaling cascade maximizes the ST for the duration of signal transduction. We introduced function *L* using the undetermined parameters *α* and *β* as follows: $L =X \sum\limits_{{{j} }} {p_{j}\Delta I_{j} } + \alpha \sum\limits_{{j}} {p_{j} } + \beta X\sum\limits_{{{{j}} }} {p_{j} \tau_{j} }$. Subsequently, we obtained $\partial L{/}\partial p_{j} = X\left( {{\text{log}}\;p_{j} {/}p_{j}^{st} } \right) + \beta X \tau_{j} + \alpha + X,$ $\partial L{/}\partial X = \sum\limits_{{j }} {\left( {p_{j} {\text{log}}\;p_{j} {/}p_{j}^{st} + \beta \, p_{j} \tau_{j} } \right)}$. To determine *α* and *β*, ∂*L/*∂*p*_*j*_ and ∂*L/*∂*X* were set to zero, which yielded $-X= \alpha$^20^ and

6 $$\frac{1}{{\tau }_{j}}\mathrm{log}\frac{{p}_{j}}{{{p}_{j}}^{st}}=-\beta$$

Comparing Eqs. (5) and (6), we deduced that *β*_*j*_ = − *β*, indicating that each molecular STR is independent of the signal molecule type number, *j*. Therefore, when the ST is maximized, STRs tend toward a constant value, and we predicted that the STR of each molecule in the EGFR cascade would have a similar value^21,22^.

### A431 stimulation with EGF

To examine whether STRs tended toward similar values, we stimulated cultured A431 cells with EGF^13^. The A431 cells were then collected at different times for protein extraction. The phosphorylation molecule ratio, *p*_*j*_*/p*_*j*_^*st*^, in Eq. (4) was obtained from the fluorescence intensity of an antibody microarray against phosphorylated signal molecules in the extract. For example, we sampled the cultured cells at *t* = 0, 15, 20, 45, 60, and 90 min after EGF stimulation (*t* = 0) and serially measured the phosphorylation ratio, *p*_*j*_*/p*_*j*_^*st*^*.* The ratio of the common time-course pattern is shown in Fig. 1A. The ratio initially exceeded 1.0 and then decreased to 1.0 during *τ*_*j*_^1^. The ratio was integrated from *t* = 0 to *τ*_*j*_, which was estimated from the plot (see S1 Text and S1 Fig.).

### Signal transduction rate in the Src-Raf1-MEK1-ERK1 cascade

The cascade network expected to be activated following EGF stimulation is shown. We applied the Bayesian statistic approach to evaluate the similarity of the STRs. The expected a posterior (EAP) values in Bayesian statistics correspond to the mean in classical statistics. We set *μ*_*j*_ representing the EAP of STR of the *j*th step (*n = *6). The STR difference (|*μ*_*j*_ − *μ*_*j*__+1_|) between the *j*th and *j* + 1th steps, effect size |*δ*_*j*_| =|*μ*_*j*_ − *μ*_*j*+1_|/*σ*_*j*_ = Δ*μ*_*j*_/*σ*_*j*_ (*σ*_*j*_; posterior standard deviation), and probability of dominance (*π*_*d*_) were calculated^23^. (1) When the *|δ*_*j*_*|* value was < 0.3, which satisfied Cohen's criteria for significant data similarity, or (2) when the *π*_*d*_ value between the two selected signal molecules (here, *j*th and *j* + 1th molecules) was > 0.4 and < 0.6, we considered that the STRs of the two molecules to have similar values. Specifically, (3) if the probability ∆π that *μ*_*j*_ > *μ*_*j*+1_ (or *μ*_*j*_ < *μ*_*j*+1_) was > 0.4 and < 0.6, the STRs were considered similar. When two of the three conditions of (1)–(3) were met, the STR at the *j*th step and the STR at the *j* + 1th step were considered similar. 

**Figure 2:**
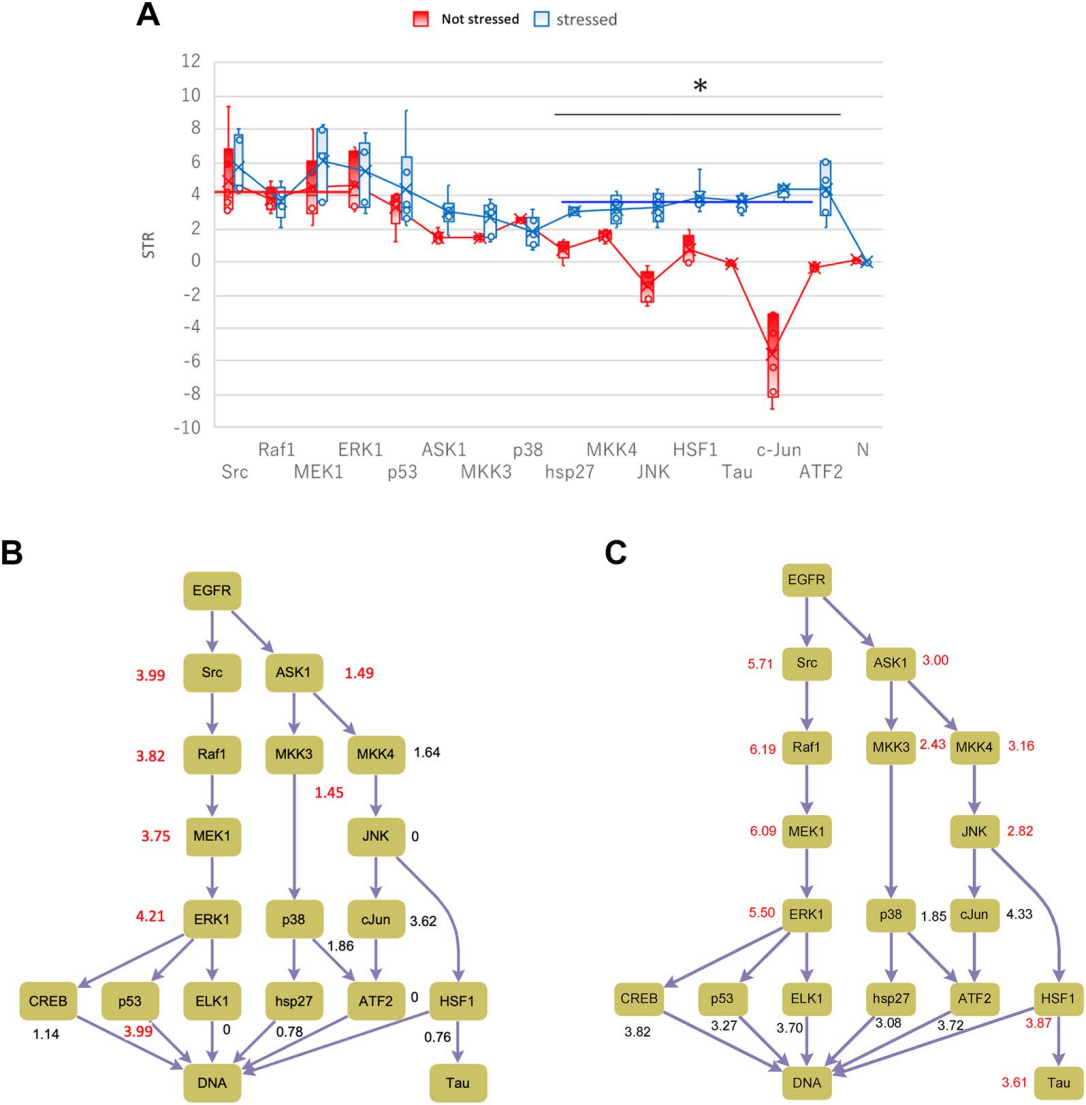
Signal transduction rate (STR) values in non-stressed and stressed cells. MAPK signal cascade in A431 cells. (**A**) Box plots of EGFR-related signaling molecules. The vertical axis represents the STR (min^−1^; *n* = 5 or 6 per protein). *N* represents the negative control using a value of the array without antibody binding. The horizontal line with the asterisk indicates significant differences between non-stressed and stressed cells (*p < 0.01, in hsp27, MKK4, HSF1, Tau, c-Jun for non-stressed vs. stressed cells). (**B**) & (**C**) Schematics of the signaling cascades during stress. (**B**) represents the non-stressed state, and (**C**) represents the stressed state. The numbers in red represent the STRs in the working cascades (|*σ*_*j*_| ≤ 0.3). *HSF1* heat shock transcription factor 1, *hsp27* heat shock protein 27^34,35^, *ATF2* activating transcription factor 2, *MKK3/4* mitogen activated protein kinase 3/4, *JNK* c-Jun N-terminal kinase.

We prepared two types of A431 cell cultures. One culture was incubated in a medium containing fetal bovine serum, whereas the other was incubated in a medium without serum to induce starvation stress. In the absence of stress, the Src-Raf1-MEK1-ERK1 (SRME) cascade was activated according to the STR values (Fig. 2A). The |*δ*_*j*_| values were < 0.3 and the *π*_*d*_ values were < 0.5. The probabilities ∆π satisfied (3) (Fig. 2B, Table 1). These data indicate that the STRs throughout the SRME cascade had similar values. Although the STR difference for ASK1-MMK3 satisfied the criteria, the cascades including steps JNK and HSP27 were not activated following stimulation because their STRs were lower (Fig. 2B, S2Data).

**Table 1:** Statistics of the expected a posteriori STR difference.

Without starvation	EAP			Probability
	STR difference	|*σ*|	*π*_*d*_	∆π
**SRME cascade**				
Src-Raf1	0.2	**0.1**	**0.5**	**0.6**
Raf1-MEK1	0.0	**0.0**	**0.5**	**0.5**
MEK1-ERK	0.5	**0.2**	**0.4**	**0.6**
ERK-p53	0.2	0.6	0.3	0.9
**AMMJ cascade**				
ASK1-MKK4	0.2	0.6	0.3	0.9
ASK1-MKK3	0.0	**0.0**	**0.5**	**0.5**

With starvation	STR difference	|*σ*|	*π*_*d*_	∆π
**SRME cascade**				
Src-Raf1	0.7	**0.1**	**0.5**	**0.6**
Raf1-MEK1	0.8	**0.1**	**0.5**	**0.6**
MEK1-ERK	0.1	**0.0**	**0.5**	**0.5**
ERK-p53	2.3	0.7	0.7	0.9
**AMMJ cascade**				
ASK1-MKK4	0.4	**0.3**	**0.4**	0.8
MKK4-JNK	0.4	**0.2**	**0.6**	0.7
JNK-HSF1	0.7	0.7	0.7	0.9
HSF1-Tau	0.0	**0.0**	**0.5**	**0.5**
ASK1-MKK3	0.3	**0.3**	**0.5**	**0.6**
MKK3-JNK	0.7	0.5	**0.6**	**0.6**

EAP, expected a posteriori, is an alternative to the mean in classical statistics. A–B represents the difference between steps A and B in the cascade shown in the left column. “STR difference” represents the difference between STR of A (STR(A)) and STR of (STR(B)). *σ* = Δμ (A − B)/*δ* (A) represents the effect size, where Δμ (A − B) represents the EAP difference between STR(A) and STR(B), and *δ* (A) represents the posterior standard deviation of STR(A). Values in the upper column “EAP” represent the EAP values of the STR difference, |*σ*|, and *π*_*d*_*.* The value under “Probability” Δπ in the upper column represents the larger probabilities of Δμ (A − B) and Δμ (B − A) (the closer the two STRs, the closer the value to 0.5). Bold letters indicate the STR differences that satisfy similarity.

For A431 cells under starvation stress, the stress-related ASK1-MKK4/MKK3-JNK (AMMJ) cascades were activated in addition to SRME, and the STR data met the similarity criteria, indicating that the STRs throughout the activated cascades had similar values (Fig. 2A,C, Table 1, S2 Data).

### t-Distributed stochastic neighbor embedding (t-SNE) analysis

To illustrate the stress effect on the activation of signal cascades, t-distributed stochastic neighbor embedding (t-SNE) analysis was performed. t-SNE transforms high-dimensional data into two dimensions and visualizes relationships between high-dimensional data using dimensionality reduction algorithms^24^. In this research, the distance distribution of a six-dimensional vector, whose elements were the STR numerical data of six replicated measurements of phosphorylation, were used. These vector data were converted to two-dimensional data that matched the distance distribution of the vector data as well as possible.

**Figure 3:**
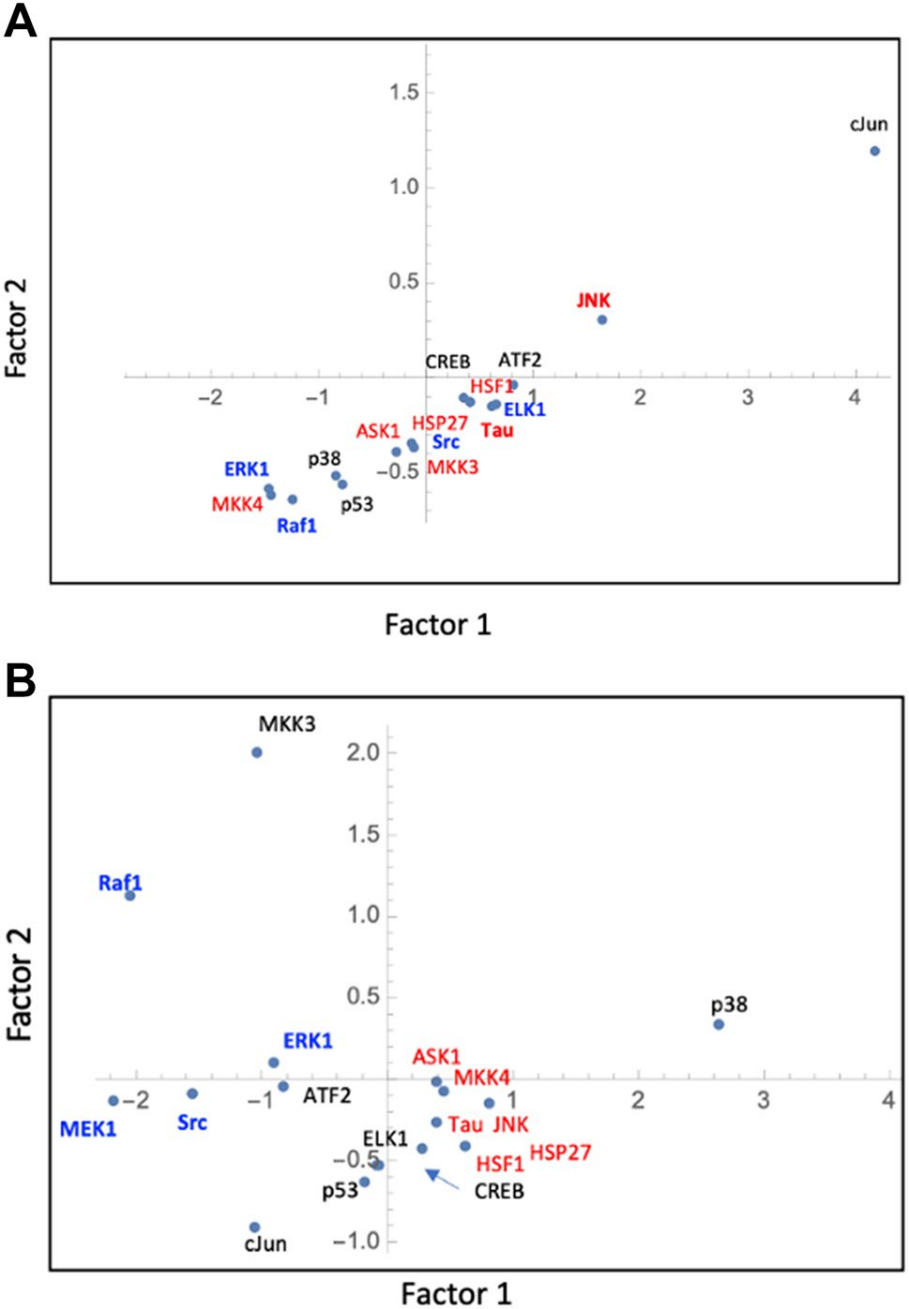
Dimension-reduction plot in the signal cascade in non-stressed (**A**) and stressed A431 cells (**B**). Blue letters represent the signal molecules in the SRME cascade, and MEK1 is out of the plot (− 2.73839, 3.19641) in (**A**). Red letters represent the signal molecules in stress-related AMMJ.

In the absence of stress, SRME and AMMJ cascade molecules showed a linear trend distribution (Fig. 3A), indicating that the STRs of the molecules participating in SRME and AMMJ were weakly similar. Under stress conditions, the AMMJ molecule STRs clustered separately from those of the SRME molecules, indicating that the AMMJ molecule STRs were similar (Fig. 3B). The clustering can be interpreted by the signal transduction cascade induced by stress. These different topologies of the plots indicate that STR-based numerical analysis effectively distinguishes cell states.

## Discussion

The KLD-based method provides a theoretical framework for quantifying signal transduction. Our experimental results indicate that the STR of each signal molecule in the cascade approached similar values when the cascades were activated^4^. The results also suggest that the cascade was activated to maximize the ST per signal. ST maximization suggests that the signal cascade does not allow for signal redundancy and that signaling transduction in A431 cells adopts a strategy that sends as many signals as possible. Another strategy is prioritizing signal accuracy that allows for redundancy. For example, sensory adaptation systems prioritize signal accuracy^25^.

Moreover, the STR analysis provides essential data on cell status. EGF stimulation in a stable state before stress mainly activates the SRME cascade, which is thought to be mainly involved in cell proliferation; however, stress-induced activation of response AMMJ cascades involving MKK3 and MKK4 was not evident. Under starvation stress, EGF stimulation activated the AMMJ cascade, as evidenced by the plots of STR similarity data and t-SNE.

Signal transduction pathways are composed of multiple steps, some of which may be phosphorylation reactions of unknown signaling molecules. In this case, if a molecule STR is similar to the STR of molecules in a given cascade, the molecule may be responsible for that unknown step in the cascade as a signal molecule. For example, the Tau transcription factor showed similar STR values at approximately 3.0 min^−1^. HSF1 contributes to Tau phosphorylation^26,27^ (Fig. 2A,C); for the HSF1-Tau step, |*δ*_*j*_| satisfied the STR similarity criteria. Therefore, STRs throughout the ASK1-MKK4-JNK and HSF1-Tau cascade had similar values, suggesting that Tau phosphorylation occurs downstream of the ASK1-MKK4-JNK cascade^28^ (Fig. 2A,C, S2 Data). MKK3 and MKK4 cooperate^29^, and MKK3 can modulate JNK phosphorylation^30^. In this study, MKK4 and JNK showed similar STRs, suggesting MKK4 may participate in JNK phosphorylation in cooperation with MKK3. Thus, STR similarity aids in identifying new steps and signal molecule cooperation in signal cascades.

We used a minimal EGF dose to measure phosphorylation. Stimulation with a high EGF dose causes excessive and non-specific cell responses in non-EGFR cascades, such as the c-Met^31^ and protooncogene c-kit^32^ receptor-related cascades. Therefore, it remains unclear how cells react to strong in-vivo stimuli, and the ligand dose limits the applicability of our results. Further studies are needed to verify our findings and investigate the stimulation range for which the cell uses a maximization strategy.

## Conclusions

The signal transduction system in A431 cells prioritizes the transduction of as many signals per unit time as possible, which may be an essential property of cell signal transduction. In addition, our approach based on the ST and STR is a promising method for identifying novel active cascades and investigating cellular responses.

## Methods

### Cell culture

The A431 human cell line derived from epidermoid carcinoma was obtained from RIKEN BioResource Research Center (Tsukuba, Japan). For the first experiment, A431 cells (0.6 × 10^5^) in 6 mL of Dulbecco's Modified Eagle's Medium (Nacalai Tesque, Kyoto, Japan) containing 10% fetal bovine serum were cultured in a 5% CO_2_ atmosphere at 37 °C for five days. EGF (100 ng/mL; Cell Signaling Technology, Danvers, MA, USA) or phosphate-buffered saline as a negative control was added to the cultures and incubated for 0 (untreated), 15, 30, 45, 60, 120, and 180 min. The total cell protein was extracted using a radioimmunoprecipitation buffer containing a protease inhibitor and phosphatase inhibitor cocktail (Nacalai Tesque). The cell extract was purified using an antibody array assay kit (Full Moon BioSystems, Inc., Sunnyvale, CA, USA).

### Antibody array assay

Antibody arrays were purchased from Full Moon BioSystems (PMK185 and PEG214). Biotinylation of the proteins and conjugation and detection by Cy3-streptavidin (PA43001; GE Healthcare Life Science, Little Chalfont, UK) were performed using an antibody array assay kit (Full Moon BioSystems) according to the manufacturer’s instructions. Protein samples (60 μg) were used in the antibody array assay. To calculate the STR using Eq. (5), the detection assay was performed six times independently following the addition of EGF to the cultures (50 ng/mL using the PEG214 array and 100 ng/mL using the PMK185 array). The mean fluorescent intensity (± standard deviation) was utilized to calculate the transduction characteristics. The antibody arrays were scanned using a SureScan Microarray Scanner (G2565CA Microarray Scanner System; Agilent Technologies, Santa Clara, CA, USA), after which the acquired image data were analyzed. The signal intensity was normalized by dividing the result by the negative control values from the array. Each value for phosphorylated proteins was divided by the respective value for unphosphorylated proteins at 0, 15, 30, 45, 60, 120, and 180 min. Finally, the results were divided by the value at 0 min to calculate the increase in phosphorylated molecules. The coefficient of variation for six replicates was < 0.1^33^.

### Phosphorylation plot

Before adding EGF, A431 cells were cultured with or without serum starvation. After adding EGF to the medium, the cells were collected at different times (0, 15, 30, 45, 60, 120, and 180 min) to extract proteins. Next, the change in the phosphorylation ratio ∫_0_^*τj*^ (*X*_*j*_^*st*^ + Δ*X*_*j*_) *dt*/∫_0_^*τj*^* X*_*j*_^*st*^* dt* of each phosphorylated protein was plotted by measuring the fluorescence levels of the antibodies against the protein in the microarray as the amount of phosphorylated *X*_*j*_. The ratio was estimated by measuring the relative fluorescence intensity of the antibody microarray. The time required for the phosphorylation level to return to that observed before adding EGF was considered the signal duration, *τ*_*j*_. Specifically, assuming that the increased level of phosphorylated signal molecules at measurement time *t*_*1*_ was *ΔX*_*1j*_ and that the increased level of phosphorylated signal molecules at measurement time *t*_*2*_ was *ΔX*_*j2*_, then *τ*_*j*_ is given by the following equation (S1 Fig.):

7 $$\tau_j = \frac{{t_{2} \Delta X_{1j} - t_{1} \Delta X_{2j} }}{{\Delta X_{1j} - \Delta X_{2j} }}$$

Finally, the STR during the phosphorylation of each protein was calculated by dividing the logarithm of the ratio, *p*_*j*_/*p*_*j*_^*st*^, by *τ*_*j*_ (S1 Data).

### Noise level evaluation

As a negative control, we measured the fluorescence intensity of the microarray spot to which the non-specific antibody was bound. Using the negative control data (S2 Data), the noise contribution to the maximal STR was estimated as 0.10 min^−1^.

### Statistics

Bayesian statistics were applied to analyze the STR at each step. For the Markov-chain Monte Carlo method, we used the R program (https://www.r-project.org/; Rstan; http://mc-stan.org/users/interfaces/rstan) to generate random numbers to obtain a posterior predictive distribution of the average entropy change rate. The R_hat_ value was 1.0 (R_hat_ is a Markov-chain Monte Carlo convergence index, and the series of values is generally considered as "converged" when R_hat_ ≤ 1.1). In this study, R_hat_ ≤ 1.1 in the calculation.

Measured STR values from five to six experiments were input to the algorithm. Outliers were excluded; however, a minimum of five replicate values was used.

### Two-dimensional reduction analysis

We conducted a two-dimensional reduction analysis using the Mathematica software (Wolfram, IL, USA). The raw data are shown in S2 Data. The STR data were represented by a four-dimensional vector, excluding the minimum and maximum data as outliers. The Mathematica core was as follows: vectors = {{*a*_11_, *a*_12_, *a*_13_, *a*_14,_ *a*_15_}, (*a*_21_, *a*_22_, *a*_23,_ *a*_24_, *a*_25_),…,{*a*_*j1*_*, a*_*j2*_*, a*_*j3*_*, a*_*j4*_*, a*_*j5*_},…}; DimensionReduce[vectors, 2]. {*a*_*j*1_, *a*_*j*2_, *a*_*j*3_, *a*_*j*4_, *a*_*j*5_} indicates the list of the mean of the STR of the *j*th molecule. The vertical and horizontal axes represent the relative topological distances determined in the dimension reduction analysis. The distribution pattern indicates the relationship between the STR data, and clustering of the data in the plots indicates correlation of those data.

## Supplementary Information

Supplementary Information 1.

Supplementary Information 2.

## Data Availability

The datasets generated and/or analysed during the current study are available from the corresponding author upon reasonable request.
