# Supplementary material for: Epidermal growth factor receptor cascade prioritizes the maximization of signal transduction
Source: Sci Rep. 2022 Oct 10;12:16950. doi: 10.1038/s41598-022-20663-0 (PMC9550784; doi:10.1038/s41598-022-20663-0)
Supplement: Supplementary file 1 — Supplementary Information 1. [file 41598_2022_20663_MOESM1_ESM.docx]

**Supplementary Materials**


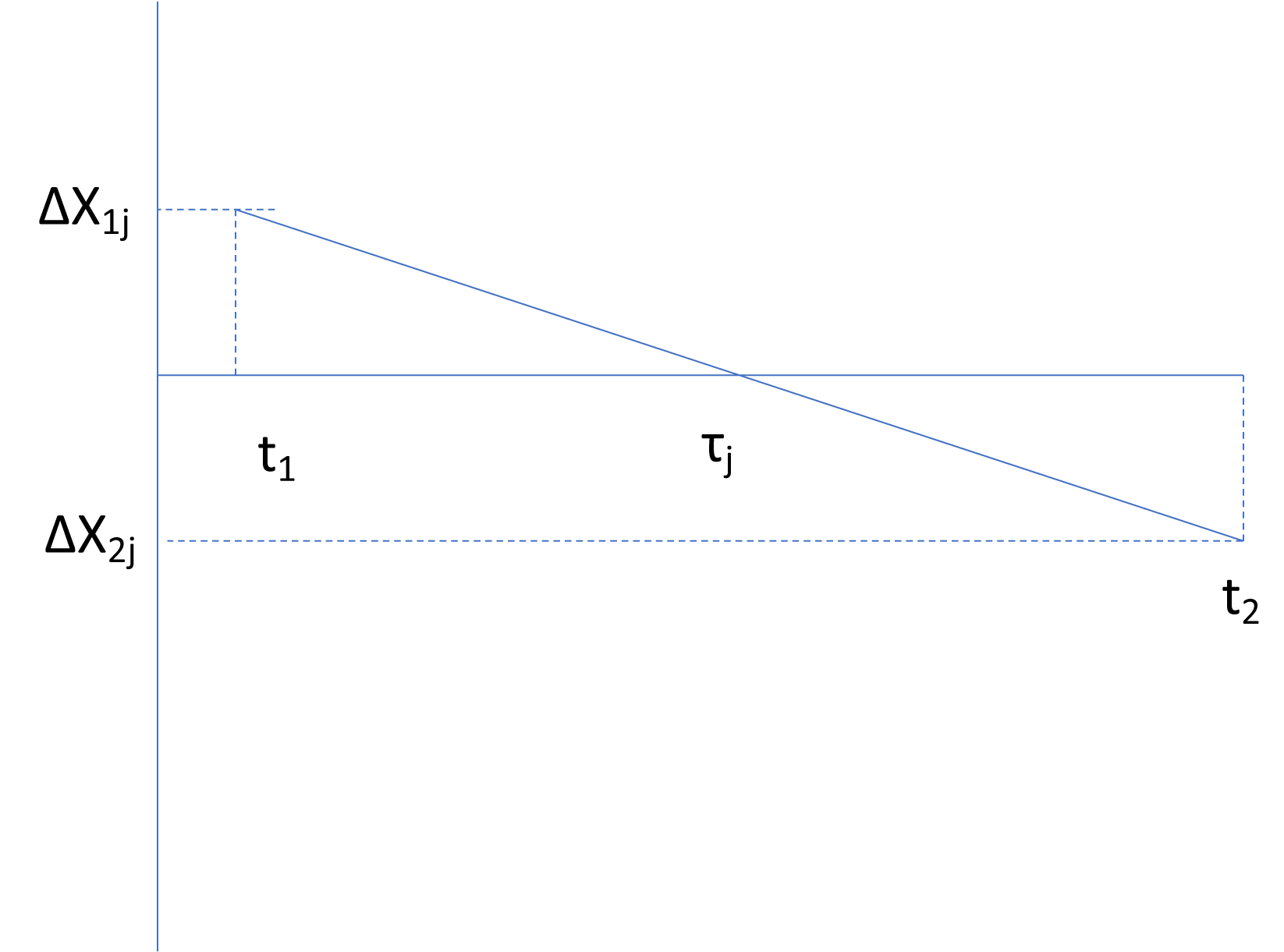


**S1 Fig. Estimation of the duration *τ_j_*.** Plot showing that Δ*X_j_* of the *j*-th molecule becomes 0 at *τ_j_* between the observation times *t_1_* and *t_2_*. Δ*X_1j_* and Δ*X_2j_* represent the increases in *t_1_* and *t_2_*, respectively.

**Supplementary data legend**

**S1 Data (separate file). Raw data used in the antibody array of non-stressed and stressed cells.**

Calculation of the average signal transduction rate (STR) for a single step, including signaling molecule phosphorylation following EGF stimulation in starved A431 cancer cells. The integral area at *t =* 0 was set to 1.0 for normalization. The ratio of the signal intensity to the value at *t =* 0 was calculated for area integration*.* The antibodies are listed in alphabetical order. "0–180 min" indicates the time elapsed after stimulation. “Phosphorylation ratio” represents the ratio (*X_j_^st^*+Δ*X_j_*)/*X_j_^st^*. “β_j_” represents the ratio of phosphorylation after EGF stimulation to phosphorylation before stimulation, ∫*_t_* _= 0_ *^t =^* (*X_j_^st^* + Δ*X_j_*) *dt*/∫*_t_* _= 0_ *^t = τ^ X_j_^st^ dt* in Eq. (5) “Log β_j_” represents the logarithmic value of the βj based on the Napier number. “*τ_j_*” indicates the signal duration of each signaling molecule. The coefficient of variation for six replicates of each ratio was <0.1 for 180 min. STR (/min) was calculated as log βj/*τ_j_* × 10^3^. A431 cells were stimulated with 50 ng/mL EGF after starvation stress. A431 cells were stimulated with 100 ng/mL EGF with starvation stress (+) or without starvation stress (–).
